# Murine Macrophages Modulate Their Inflammatory Profile in Response to Gas Plasma-Inactivated Pancreatic Cancer Cells

**DOI:** 10.3390/cancers13112525

**Published:** 2021-05-21

**Authors:** Aydar Khabipov, Eric Freund, Kim Rouven Liedtke, Andre Käding, Janik Riese, Julia van der Linde, Stephan Kersting, Lars-Ivo Partecke, Sander Bekeschus

**Affiliations:** 1 Department of General, Visceral, Thoracic and Vascular Surgery, Greifswald University Medical Center, Ferdinand-Sauerbruch-Str., 17475 Greifswald, Germany; aydar.khabipov@med.uni-greifswald.de (A.K.); eric.freund@inp-greifswald.de (E.F.); andre.kaeding@med.uni-greifswald.de (A.K.); janik.riese@stud.uni-greifswald.de (J.R.); julia.vanderlinde@med.uni-greifswald.de (J.v.d.L.); stephan.kersting@med.uni-greifswald.de (S.K.); Ivo.Partecke@med.uni-greifswald.de (L.-I.P.); 2 ZIK *plasmatis*, Leibniz Institute for Plasma Science and Technology (INP Greifswald), Felix-Hausdorff-Str. 2, 17489 Greifswald, Germany; 3 Department of Trauma and Orthopedic Surgery, Schleswig-Holstein University Medical Center, Arnold-Heller-Straße 3, 24105 Kiel, Germany; kim.liedtke@med.uni-greifswald.de; 4 Department of General, Visceral and Thoracic Surgery, Schleswig Helios Medical Center, St. Jürgener Str. 1-3, 24837 Schleswig, Germany

**Keywords:** CAP, cold atmospheric pressure plasma, cold physical plasma, kINPen, PDA6606, plasma medicine, metastasis, tumor microenvironment (TME)

## Abstract

**Simple Summary:**

Pancreatic cancer is a devastating disease with high mortality. The cancer is characterized by a dynamic and immunosuppressive tumor microenvironment (TME) with high numbers of macrophages. Gas plasma technology was previously suggested as a promising new tool in oncology and pancreatic cancer treatment. However, it is unclear how gas plasma-treated pancreatic cancer cells affect the phenotype and inflammatory profile of macrophages. Besides profound antitumor effects of gas plasma-exposed tumor cells, we identified in such co-cultures unique signatures of both pro- and anti-inflammatory mediators being secreted at elevated levels. These responses might be beneficial as they promote neither overshooting inflammation and metastasis nor immunosuppression, fueling tumor growth as a known consequence of anti-inflammation.

**Abstract:**

Macrophages and immuno-modulation play a dominant role in the pathology of pancreatic cancer. Gas plasma is a technology recently suggested to demonstrate anticancer efficacy. To this end, two murine cell lines were employed to analyze the inflammatory consequences of plasma-treated pancreatic cancer cells (PDA) on macrophages using the kINPen plasma jet. Plasma treatment decreased the metabolic activity, viability, and migratory activity in an ROS- and treatment time-dependent manner in PDA cells in vitro. These results were confirmed in pancreatic tumors grown on chicken embryos in the TUM-CAM model (in ovo). PDA cells promote tumor-supporting M2 macrophage polarization and cluster formation. Plasma treatment of PDA cells abrogated this cluster formation with a mixed M1/M2 phenotype observed in such co-cultured macrophages. Multiplex chemokine and cytokine quantification showed a marked decrease of the neutrophil chemoattractant CXCL1, IL6, and the tumor growth supporting TGFβ and VEGF in plasma-treated compared to untreated co-culture settings. At the same time, macrophage-attractant CCL4 and MCP1 release were profoundly enhanced. These cellular and secretome data suggest that the plasma-inactivated PDA6606 cells modulate the inflammatory profile of murine RAW 264.7 macrophages favorably, which may support plasma cancer therapy.

## 1. Introduction

The 5-year survival rate in pancreatic cancer patients has stagnated for decades [1], and incomplete surgical resection [2], inherent or acquired drug resistance [3], metastasis [4], and a cancer growth-supportive tumor microenvironment (TME) [5], among others, are related to the poor therapeutic outcomes. Pancreatic ductal adenocarcinoma (PDA) is characterized by skewing the polarization of local macrophages towards an M2 phenotype, called tumor-associated macrophages (TAM), which in its essence promotes cancerogenesis, e.g., via the release of several factors [6]. The factors, mostly chemokines and cytokines that are also released by PDA cells, shape the TME in favor of growth, and include, for instance, transforming growth factor (TGF)β that dampens immunity and promotes cellular growth via Smad-mediated signaling following activation of serine/threonine kinase-linked receptors [7]. Several other factors, such as vascular endothelial growth factor (VEGF) spurring angiogenesis, as well as redox signaling and metabolism [8], amplify cancer infiltration and metastasis [9,10,11].

A novel concept of targeting cancer is the utilization of gas plasma technology that generates vast amounts of short-lived reactive oxygen and nitrogen species (ROS/RNS), simultaneously [12]. These ROS/RNS can be released onto tumor cells and tissues and are capable of inflicting both terminal and transient damage, not only augmenting cell death [13] but also modulating inflammatory pathways in the microenvironment [14]. The work of others, as well as our own work, has previously suggested a putative role of plasma treatment against pancreatic cancer [15,16,17], especially as a post-surgical add-on procedure of resection margins. Given the importance of macrophages in the pancreatic TME [18] and cell migration, the current study aimed to investigate both aspects following plasma treatment using the safe and accredited argon plasma jet kINPen [19]. Plasma treatment was found to dampen tumor cell migration and their ability to hijack co-cultured macrophages to release tumor-supportive factors, such as VEGF, supporting the idea of plasma exposure as an adjuvant treatment during surgery.

## 2. Results

### 2.1. Plasma Exposure Inhibited Pancreatic Cancer Cell Metabolism and Migration In Vitro

**Figure 1:**
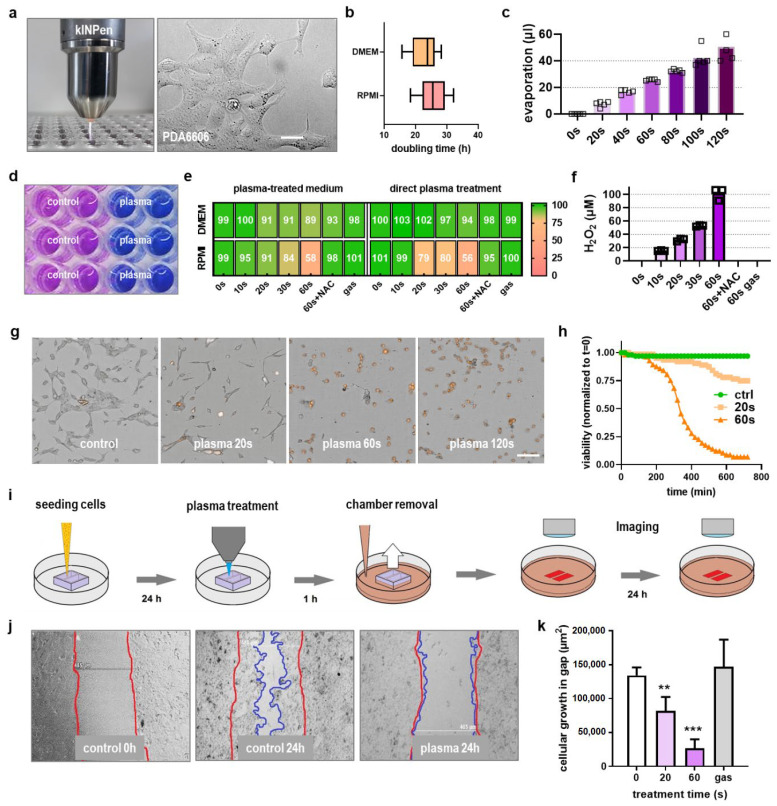
Cytotoxicity and migration in pancreatic cancer cells in vitro. (**a**) Macroscopic image of the atmospheric pressure argon plasma jet kINPen (left) and microscopy image of PDA6606 murine pancreatic cancer cells used in this study (right); (**b**) doubling time of PDA6606 cells in fully supplemented DMEM and RPMI culture medium; (**c**) evaporation of liquid during gas plasma treatment of cultures; (**d**) representative macroscopic image of the resazurin-based assay on cellular metabolism for control and 60 s plasma-treated cells at 24 h; (**e**) metabolic activity of cells following exposure to direct plasma treatment or plasma-treated cell culture medium (in % of values normalized to untreated controls); (**f**) H_2_O_2_ generation during plasma treatment of cell culture medium; (**g**) representative brightfield and propidium iodide (PI, orange) images of PDA6606 12 h post-exposure; (**h**) kinetic of relative number of dead cells following plasma treatment; (**i**) scheme of the migration assay; (**j**) representative brightfield images of untreated and plasma-treated cells of the migration assay, the red and blue lines mark the boarders at 0 and 24 h, respectively; (**k**) quantification of absolute cell areas in untreated and plasma-treated cells. Scale bars are 20 µm (**a**), 50 µm (**g**), and 485 µm (**j**). ** = *p* < 0.01; *** = *p* < 0.001; NAC = n-acetylcysteine; gas = exposure to argon with plasma-off.

In the current study, the atmospheric pressure argon plasma jet kINPen was used to treat murine PDA6606 cells (Figure 1a). The aim was to investigate the cytotoxic action of direct exposure to the kINPen plasma to these cells and their migration first before analyzing the functional consequences in co-cultures with murine macrophages in a second step. The pancreatic cancer cells’ proliferation was higher in RPMI over DMEM cell culture medium (Figure 1b), leading to the utilization of the former over the latter for subsequent experiments. During in vitro plasma treatment of cultures, the liquid evaporates, which was compensated for by predetermination of evaporation and supplementation with double-distilled water after exposure (Figure 1c). Subsequently, the metabolic activity of plasma-treated PDA6606 cells was determined (Figure 1d). Quantification and normalization of metabolic activity 24 h post-exposure revealed a treatment time-dependent decrease to a similar extent when analyzing both freshly plasma-treated cell culture medium and direct plasma treatment conditions (Figure 1e). The effects of kINPen plasma-treated medium had been outlined before in detail [20], but this procedure is not accredited compared to direct plasma treatment, which is why the latter was used throughout this study. The ROS/RNS scavenging antioxidant n-acetylcysteine (NAC) abrogated cytotoxic plasma effects in both treatment conditions, while exposure to the argon gas alone (plasma: off) had no effect at all. The short-lived ROS released from plasmas deteriorate to long-lived oxidants such as hydrogen peroxide (H_2_O_2_), which was generated in cell culture medium in a treatment time-dependent manner (Figure 1f). To study whether the reduced metabolic activity was due to cell growth inhibition or terminal cell death, propidium iodide (PI) staining was used, marking terminally dead cells (Figure 1g). Kinetic analysis of the percentage of viable cells showed a marked decline with increasing plasma treatment times (Figure 1h). Subsequently, a migration chamber assay was deployed (Figure 1i) to investigate cellular motility following plasma treatment, which was found to be decreased (Figure 1j). Quantitative image analysis of the absolute cellular growth area showed that this decline was significant (Figure 1k).

### 2.2. Plasma Exposure Inhibited PDA Growth via Apoptosis in the TUM-CAM Model In Ovo

**Figure 2:**
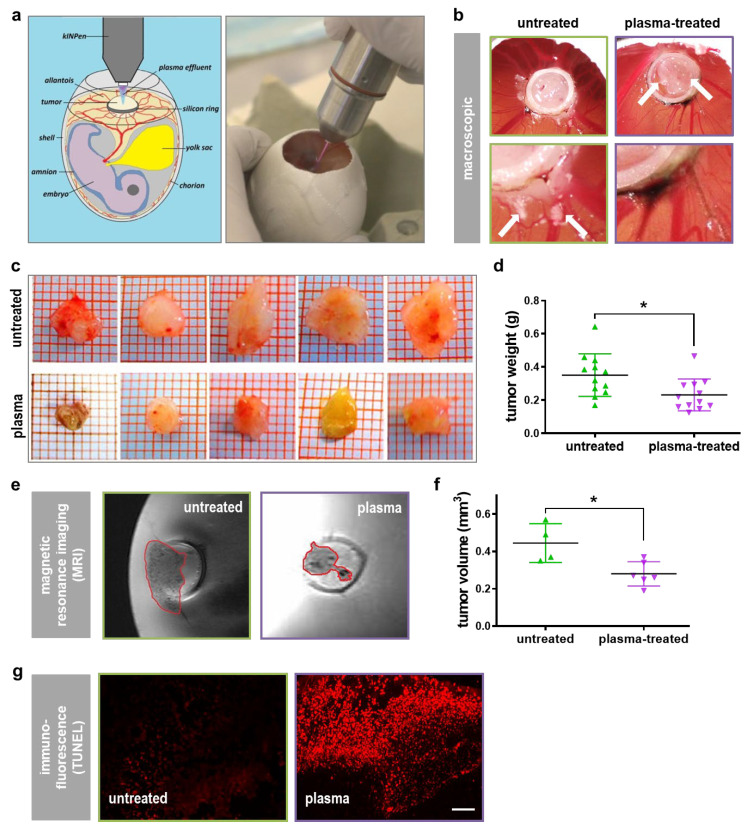
Cytotoxicity in pancreatic cancer cells in ovo. (**a**) Scheme of the in ovo tumor model (left) and macroscopic image of the tumor plasma treatment in ovo; (**b**) macroscopic images of untreated and plasma-treated tumors in ovo (top) and close-up images showing the presence and absence of surrounding tumor lesions, respectively; (**c**) macroscopic images of five explanted in ovo tumors from the untreated and plasma-treated groups, respectively; (**d**) tumor weight of explanted in ovo tumors; (**e**) representative MRI of an untreated and plasma-treated tumor; (**f**) volumetric quantification of tumors grown in ovo using MRI; (**g**) representative immunofluorescence microscopy images showing TUNEL^+^ (apoptotic) cells. Scale bar is 200 µm; * = *p* < 0.05.

To demonstrate the anticancer effect of direct plasma treatment in vascularized three-dimensional tumors, PDAs were grown on the chorion-allantois-membrane (CAM) of chicken embryos (Figure 2a). Macroscopically, plasma treatment reduced tumor growth and metastatic tumor outgrow lesions extending outside the primary tumor area (Figure 2b). To quantify growth reduction, tumors were explanted (Figure 2c), and plasma exposure significantly decreased total PDA tumor weight grown in the TUM-CAM model (Figure 2d). In another experiment, TUM-CAM-bearing chicken embryos were subjected to magnetic resonance imaging (MRI) to calculate the tumor volume (Figure 2e). The quantification revealed a significant size reduction in the plasma treatment conditions (Figure 2f). The reduction of tumor mass was associated with a marked elevation of apoptotic cells in the cancer tissue (Figure 2g), as evident using TdT-mediated dUTP-biotin nick end labeling (TUNEL).

### 2.3. Macrophages Recognized Plasma-Treated PDA6606 Cells

**Figure 3:**
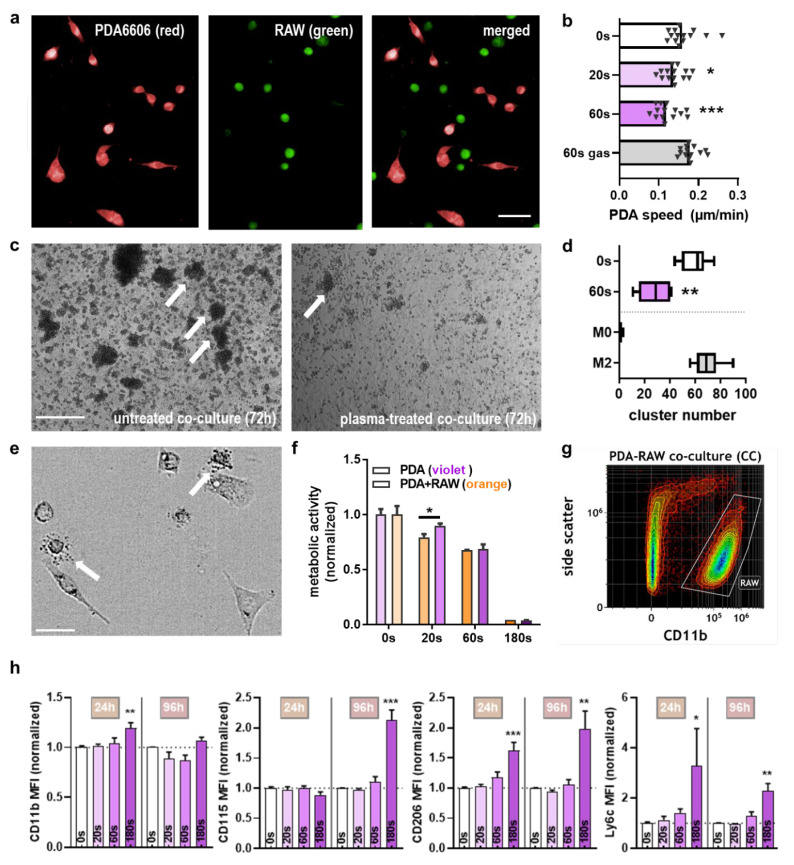
Co-culture of pancreatic cancer cells with macrophages and their activity. (**a**) Live-cell imaging of DiL-stained PDA6606 (PDA) cells (red), CFSE-labeled RAW 264.7 (RAW) macrophages, and overlay; (**b**) quantification of tumor cell migration over 20 h; (**c**) representative brightfield images of untreated co-cultures of PDA and RAW cells leading to clustering of the latter (arrow) as well as plasma-treated co-cultures showing less clustering at 72 h post-exposure; (**d**) quantification of clusters and comparison to RAW cells alone (M0) and M2-skewed RAW cells per field of view; (**e**) representative brightfield images of granular release from RAW cells (arrow); (**f**) metabolic activity of untreated and plasma-treated PDA cells at the plate bottom with RAW cells co-cultured in a transwell system on top, with a pore size that did not allow for transmigration, metabolic activity was measured of PDA cells alone after removing the transwells harboring the RAW cells at 24 h; (**g**) representative flow cytometry density dot-plot showing the discrimination of RAW cells from PDA cells in co-culture assays using CD11b fluorescently labeled antibodies; (**h**) quantification and normalization of the mean fluorescent intensities (MFI) over several surface markers on RAW cells co-cultured with PDA cells for 24 and 96 h. Scale bars are 25 µm (**a**), 250 µm (**c**), and 20 µm (**e**). Gas = exposure to argon with plasma-off; * = *p* < 0.05, ** = *p* < 0.01; *** = *p* < 0.001.

Plasma treatment inflicted damage and halted PDA6606 growth, so the next purpose was to investigate the murine macrophages’ (RAW 264.7 cells) response in this context. To this end, macrophages were added to plasma-treated PDA cells (Figure 3a), and the migration speed of PDA cells was quantified. Similar to the consequences of plasma treatment of PDA cells alone, a reduction in cell motility was also retained in the presence of macrophages (Figure 3b). PDA cells can skew macrophages towards an M2 phenotype, which correlates with a clustering behavior of macrophages (Figure 3c). Plasma exposure of PDA and subsequent co-culture with macrophages significantly reduced the number of macrophage clusters compared to untreated PDA cells (Figure 3d). During co-cultures and microscopy analysis, macrophage degranulation was observed (Figure 3e). Hence, we wondered whether macrophages co-cultured with PDA cells in a transwell system, which only allows for an exchange of soluble factors but not any physical interaction or transmigration, would be capable of affecting PDA growth. Indeed, the addition of macrophages to 20 s plasma-treated PDA cells enhanced the toxic effect, while for the other exposure times, an additive effect was not observed (Figure 3f). At this point, we had also introduced a 180 s plasma treatment time, which was supposed to inactivate the large majority of PDA cells to investigate the consequences of this condition in macrophages. A good measure of the inflammatory phenotype of macrophages is flow cytometry examining cell surface markers representative for polarization and differentiation. As these processes take time, macrophages were co-cultured with untreated or plasma-treated PDA cells and investigated at both 24 and 96 h post-plasma exposure of PDA cells. Using flow cytometry, macrophages were discriminated from PDA cells using their surface marker CD11b (Figure 3g), and a modest but significant increase was observed for this marker at 24 h post 180 s plasma treatment of PDA (Figure 3h), suggesting some degree of activation. For CD115, which is associated with an M1 phenotype, a significant increase was observed for the same condition at 96 h, while the M2 marker CD206 was significantly elevated in the 180 s plasma treatment time conditions for both 24 and 96 h. Ly6C, which is associated with activation and differentiation, was increased in those conditions as well. The shorter, less toxic plasma treatment times had no significant effect when normalizing the surface marker MFIs against the respective co-culture with untreated PDA cells.

### 2.4. Chemokine and Cytokine Profiles of Mono- and Co-Cultures with Plasma Treatment

**Figure 4:**
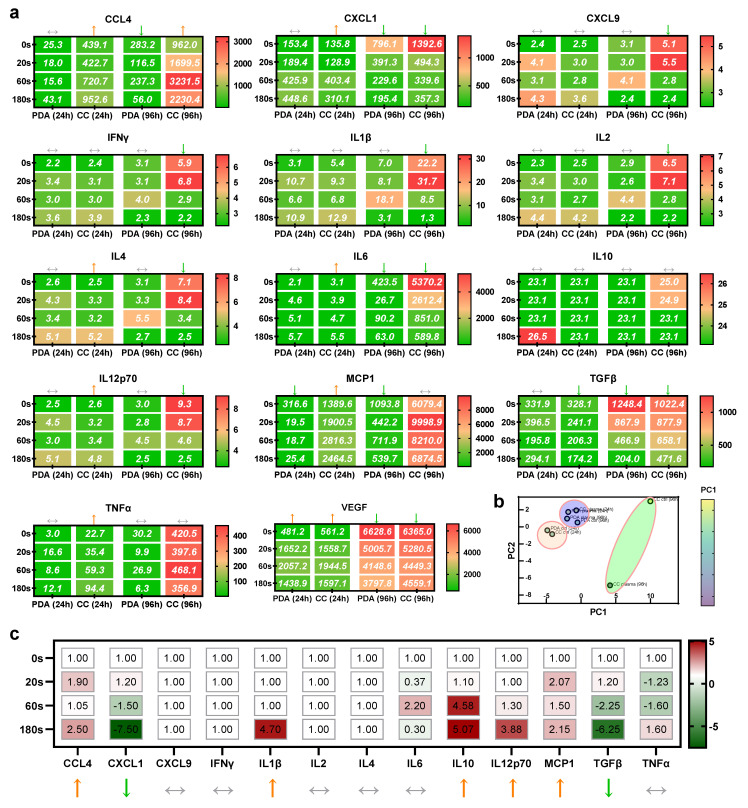
Chemokine and cytokine profiles of co-cultures. (**a**) Mean absolute concentrations of 14 different chemokines and cytokines analyzed in supernatants of untreated and plasma-treated PDA6606 (PDA) monocultures as well as RAW 264.7 (RAW) co-cultures (CC) collected at 24 and 96 h, where arrows indicate significant tendencies and values in boxes represent absolute concentrations in pg/mL; (**b**) principal component analysis of all chemokines and cytokines and reducing to untreated and plasma-treated samples, showing three distinct clusters of similarity for 96 h co-cultures (green), 24 h untreated conditions for the monoculture and co-culture (orange), and all other samples (violet); (**c**) supernatant chemokine and cytokine release normalized to untreated controls (0 s) of PDA and RAW cells co-cultured in transwell systems over 24 h. Arrows indicate non-significant (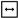) or significant trend for linearity (↑ or ↓). CCL = CC-chemokine ligand; CXCL = (C-X-C motif) ligand; IL = interleukin; IFN = interferon; MCP = macrophage-chemoattractant protein; TGF = transforming growth factor; TNF = tumor necrosis factor; VEGF = vascular endothelial growth factor.

To understand the secretion profiles of the mono and co-cultures at 24 and 96 h, and the transwell system of co-cultures at 24 h, more than a dozen chemokines and cytokines were analyzed. For the former setup, several targets were found to be modulated by the plasma treatment. In PDA cells alone at 24 h, an increase of VEGF and a decrease of MCP1 was measured (Figure 4a), while at 96 h, a decrease of CCL4, CXCL1, IL6, MCP1, TGFβ, and VEGF was found, with no target being investigated showing an increase with plasma exposure at this incubation time. For the co-cultures at 24 h, an increase of CCL4, CXCL1, IL4, IL6, IL12p70, MCP1, and TNFα was determined, while TGFβ was decreased. For the co-cultures at 96 h, elevated levels were found for CCL4, and a decrease was measured for CXCL1, CXCL9, IFNγ, IL1β, IL2, IL4, IL6, IL12p70, TGFβ, and VEGF. Arrows indicate trends that were significant when analyzed using one-way analysis of variances. The samples were segregated based on untreated versus plasma-treated and analyzed using principal component analysis (PCA) to aggregate this extensive dataset. Against the first main principal components, three distinct clusters appeared (Figure 4b). The first cluster (orange) harbored untreated samples of mono- and co-cultures at 24 h. A second cluster (orange), which was most distinct across both principal components, included the 96 h samples of untreated and plasma-treated co-cultures. The remaining samples constituted a third cluster (blue). The significant trends of the transwell co-cultures at 24 h were partially congruent with the trends of the direct co-cultures, e.g., for CCL4, IL12p70, MCP1, and TGFβ but not for CXCL1, IL1β, and IL10 (Figure 4c). In summary, we found a large set of significant changes induced by plasma treatment of PDA cells in macrophages known to be critical in the TME. For instance, the decrease of TGFβ is associated with less immunosuppression, VEGF is crucial for angiogenesis, and CCL4 is a pro-inflammatory chemokine.

## 3. Discussion

This study aimed at identifying the macrophage response to gas plasma-inactivated pancreatic cancer cells. Several changes in the macrophage surface marker expression and chemokine and cytokine secretion profiles suggested recognition of plasma-induced pancreatic cancer cell death and subsequent inflammatory modulation of the local microenvironment.

Plasma-mediated inactivation of pancreatic cancer cells has been shown before, which we could confirm in this study for murine PDA6606 cells. Increased cell death was previously demonstrated for the kINPen plasma jet and human pancreatic cancer cells in vitro and in ovo [21]. This was also shown for other types of plasma jets, e.g., a helium discharge that had also shown combined cytotoxicity with the 5-FU prodrug Tegafur [22]. Malign migration acts as a crucial factor for cell invasion of surrounding tissue and metastasis in further course [23,24,25]. Yet, reports on the migratory behavior of murine pancreatic cancer cells following plasma exposure are scarce but documented for other tumor cell types, such as colorectal cancer [26]. For human pancreatic cancer cells, we previously demonstrated a lack of increased migration or EMT phenotypes [21]. Our data provided evidence that cell death was dependent on the generation of reactive oxygen species (ROS) found to be released by the kINPen [27], which is in line with previous findings linking the plasma-derived ROS to ferroptotic cell death in plasma-treated pancreatic cancer cells [28]. ROS were suggested as a promising anticancer strategy since, for instance, K-Ras-mutated cancer cells such as PDA already have higher baseline levels of oxidative stress and express elevated levels of aquaporins [29], which supports H_2_O_2_ influx [30]. We and others have shown that the application of plasma-treated medium and clinically approved liquids such as saline trigger cell death in murine and pancreatic cancer cells as well [31,32]. For plasma-treated medium and plasma-treated Ringer’s lactate, in vivo studies provided evidence of the clinical efficacy of this approach [17,20,33]. Interestingly, recent work also suggested effects on inflammation and the tumor microenvironment of this treatment regimen [34,35], but how macrophages specifically perceive plasma-treated pancreatic cells has not been studied so far.

To understand this, the response of PDA cells to plasma treatment needs to be reflected first. The data obtained 96 h post-plasma treatment are of particular interest, showing PDA cells’ mid-term adaption response. For plasma-treated PDA cells alone, a significant decrease was found for CCL4, CXCL1, IL6, MCP1, TGFβ, and VEGF. Based on the analyses of 75 human pancreatic cancer tissues, Itakura and colleagues postulated in 1997 that “enhanced expression of vascular endothelial growth factor in human pancreatic cancer correlates with local disease progression“ [36]. Today, VEGF is a known pro-angiogenic factor critically spurring metastasis [37,38], and its mid-term decrease is putatively beneficial. However, the immediate response (24 h) was associated with a modest but significant increase of VEGF, which might be a result of cellular damage per se. In the clinical context, VEGF inhibitors—such as the monoclonal antibody Bevanzizumab—could be applied simultaneously with plasma treatment to tackle tumor growth by anti-angiogenic and pro-apoptotic therapies, simultaneously. The perhaps most dramatic decrease at 96 h was found for TGFβ. In cancer stages of manifested tumors, TGFβ increases the risk of metastasis [39,40]. In clinical trials with TGFβ-blocking antibodies, metastasis and infiltration were suppressed [41,42]. Therefore, inhibition of TGFβ release could act as a potential target to reduce tumor infiltration and metastasis [43]. For instance, Itraconazole, a commonly used antifungal agent, inhibits the invasion and migration of pancreatic cancer cells by suppressing TGFβ signaling [7]. This observation underlines the role of TGFβ as a target to reduce malign migration. Along similar lines, IL6 has been decreased with plasma treatment. The molecule is known to induce a pro-tumorous microenvironment [44], angiogenesis, and metastasis [45], primarily via signaling through STAT3 [46], making IL6 another attractive target for cancer therapy [47,48]. Interestingly, splenocyte supernatants obtained from an in vivo tumor model of disseminated PDA and repetitive intra-abdominal lavage via plasma-treated liquids showed decreased IL-6 levels associated with less tumor burden and increased survival [20]. CXCL1 is another crucial metastasis-promoting cytokine [49,50], which is suggested to suppress immunological defense mechanisms [38,51]. This was shown in pancreatic cancer, being intertwined by the efficacy of checkpoint immunotherapy against the PD-1/PD-L1 axis [52]. CD47 is another recently reported immune checkpoint [53] that was recently identified to be altered in response to gas plasma treatment [54]. This and the current work add to important routes for immune-relevant effects of gas plasma treatment of cancer cells. The first is immediate effects by oxidative modifications of target proteins and receptors through gas plasma-derived ROS/RNS [55,56]. This may also affect other targets of the TME, such as thiols [57,58,59] and extracellular matrix hyaluron [60], that upon plasma treatment were recently identified to show disrupted binding to its receptor CD44 [61]. The second is long-term effects on the expression levels of immune-relevant ligands and receptors, as we and others have reported recently [54,62,63,64,65,66]. Importantly, both events may combine during gas plasma exposure of cancer tissue to promote antitumor immunity [67,68]. After all, the plasma treatment might also directly affect the immune cells and their function along with chemokine and cytokine release [69,70,71]. For instance, blocking the CXCLs–CXCR2 axis in vivo was found to reduce cell invasion and migration [72], and promoted an immune-inflammatory microenvironment and prolonged survival of mice [73]. Monocyte chemoattractant protein 1 (MCP1/CCL2) and CCL4 production are linked to unfavorable prognosis [74,75] and monocyte recruitment from the periphery [76], and we have observed a decrease of these targets in plasma-treated PDA.

A putative clinical application of gas plasma technology in pancreatic cancer treatment needs to be safe, and there have been a number of studies addressing this point in the past years for the kINPen argon plasma jet. Investigating genotoxicity concerns in kINPen-treated cells, it was found in vitro and using an OECD-based assay (cytokinesis-block micronucleus assay) that gas plasma exposure is void of provoking micronuclei formation [77,78]. The phosphorylation of the histon 2A.X, a known marker for DNA double-strand breaks in radiobiology [79], is frequently observed after gas plasma exposure [80,81,82] and we could show that this was a consequence of pro-apoptotic processes rather than primary gas plasma-derived ROS/RNS directly mediating sufficient damage to promote such phosphorylation [83]. In a 1-year follow-up study in mice and human volunteers, no long-term damage, side effects, or pro-carcinogenic effects were observed [84,85]. Regarding selectivity, we recently reported in a 35-cell-line screening that the sensitivity towards gas plasma-induced cytotoxicity varies greatly within cell lines, and seems independent of the degree of malignancy [86]. Nevertheless, fibroblasts have been reported to be more resistant to gas plasma treatment than pancreatic cancer cells [16,20]. Moreover, we have recently found that gas plasma exposure promotes neither the epithelial–mesenchymal transition (EMT) in several models [87] nor metastasis and the expulsion of individual cells from 3D bulk tumors due to physical effects [21]. These results suggest an appropriate degree of confidence in the principal utilization of gas plasma technology and especially the kINPen in medicine.

Upon adding macrophages to untreated or plasma-treated PDA, some dramatic changes were observed for both tendencies and absolute levels of several mediators. What was striking is the bimodal responses observed for similar targets at the different time points. Except for the favorable decreases seen with TGFβ [88], most targets investigated in co-culture supernatants showed a significant or by-trend increase at 24 h with increasing plasma treatment times versus a significant or by-trend decrease at 96 h. Some of these changes are beneficial when it comes to non-tumor-promoting conditions, e.g., the decrease of VEGF, TGFβ, IL6, CXCL1, CXCL9, IL1β [89], and M2-macrophage-inducing IL4 [90], as well as the increase of immuno-supportive and M1-associated IL12p70 and TNFα [91]. Nevertheless, at the other time points, some of these tendencies are also associated with adverse outcomes, as discussed above, especially for CCL4 in co-cultures. However, taking into account the prominent roles of immunosuppressive targets that we found to be downregulated in the plasma-treated co-cocultures, along with the dramatic decrease of absolute concentrations (e.g., 90% for IL6 and 80% for CXCL1), our findings suggest an overall beneficial effect of plasma-treated PDA cells on murine macrophages in terms of shaping the TME. The pleiotropic IL1β was decreased in direct but not separated co-cultures, and the cytokine is involved in detrimental pancreatic cancer desmoplasia and immunosuppression [92]. IL10 was at or below the detection limit in direct co-cultures, while gas plasma exposure elevated its levels in the separated co-cultures. IL10 is clearly a tumor-promoting cytokine and is responsible for augmenting immunosuppression in the TME and providing angiogenic stimuli to facilitate tumor growth [93,94]. IL12p70 in turn was increased in direct co-cultures at 24 h and decreased at 96 h, while it was increased in separated co-cultures at 24 h. The cytokine is known to have beneficial roles in antitumor immunity [95,96], also in pancreatic cancer [97].

Considering the findings of the surface marker measurements, the plasma exposure seemed to spur a differentiation-like response over 96 h in the murine macrophages that become increasingly positive for the markers CD115, CD206, and Ly6C. Although CD206 is a known marker of M2 macrophages [98], our previous findings with polarized RAW cells showed that its absolute expression on a per-cell basis was comparable in M1 when compared to M2 cells [99]. CD115 was found to be a macrophage marker associated with a good prognosis in osteosarcoma patient samples [100]. Based on a thorough analysis of the TME in PDA tumors receiving repeated exposure to plasma-treated medium in vivo, a significant increase in the number of macrophages was observed along with a magnificent elevation of apoptotic tumor cells [20]. Similar to our findings, these intra-tumoral macrophages that had been recruited by tissue damage and possibly also some of the chemokines and cytokines described above have been educated by dying tumor cells. To our surprise, we neither found an increase of CD206 nor iNOS on macrophages in these tissues [101] being markers of M2 and M1 macrophages, respectively [102]. This suggested that final polarization to tumor-supporting M2 phenotypes or tumor-reducing M1 phenotypes might not be an immediate but continuous process under relatively stable conditions and longer timelines. This idea is well-manifested in a recent study clearly showing that tumor-associated macrophages (TAMs) [103,104] have an M1 or M2 phenotype dependent on their location and microenvironmental conditions rather than having only the trait of residing within the tumor [105]. Our findings also exemplify how delicate the time point of analysis is in evaluating immune cell function in the TME and are in line with results of both pro- and anti-inflammatory macrophage function in pancreatic cancer conditioning of these myeloid cells [106]. Especially with pancreatic cancer being a macrophage-rich tumor entity [107], we hypothesize the practical implications of our findings to be relevant in targeting resection margins following oncological surgery with plasma technology for promoting a TME that suppresses tumor recurrence of microsatellite metastases and to prolong cancer-free survival in patients, eventually.

## 4. Materials and Methods

### 4.1. Cell Culture

Murine PDA6606 (Pancreatic Ductal Adenocarcinoma, PDA) cells were kindly provided by David Tuveson (The Cancer Center, Johns Hopkins University, Baltimore, MD, USA). Murine RAW 264.7 macrophages (RAW) were purchased from ATCC (TIB-71). The cells were cultured in Roswell Park Memorial Institute (RPMI-1640; Pan-BioTech, Aidenbach, Germany) medium supplemented with 10% fetal bovine serum (Sigma, Taufkirchen, Germany) and 2% penicillin/streptomycin (Pan-BioTech, Aidenbach, Germany) under standard conditions. In one pilot study, DMEM (Dulbecco’s Modified Eagle’s Medium; Pan-BioTech, Aidenbach, Germany) was used as well. Cell counting of live cells to prepare experiments was performed using acoustic focusing flow cytometry (Applied Biosystems, Bremen, Germany). For the experiments, the cells were seeded overnight before plasma treatment to allow for adhesion. For one pilot experiment to induce M2-related cell clustering, RAW cells were exposed to IL4 and M-CSF (Miltenyi, Teterow, Germany) for three days before microscopy.

### 4.2. Plasma Treatment

The atmospheric pressure plasma jet kINPen (neoplas, Greifswald, Germany) was utilized for plasma treatment of the cells and tissues. The jet was operated at 1 MHz and total plasma-dissipated power of about 1 W to ignite a flow of argon (99.9999% purity; Air Liquide, Bremen, Germany) [108]. The plasma treatment routine was automated by attaching the kINPen to a computer-driven *xyz*-table (CNC, Lübeck, Germany) centered over each well of a 96-well plate (Eppendorf, Hamburg, Germany), with 10 µm precision in each dimension. Treatment times, i.e., the jet remaining over the center of the well for the indicated time length, were programmed in advance. The 96-well plates had an outer rim filled with double-distilled water to minimize evaporation and edge effects during long cell culture. The treatment volume was 100 µL of fully supplemented RPMI-1640 cell culture medium, if not indicated otherwise. As a control, argon gas treatment alone (plasma = off) was used in some experiments.

### 4.3. ROS Analysis

Hydrogen peroxide (H_2_O_2_) is a prime end product of plasma-derived ROS and hence a convenient measure to analyze plasma-induced ROS deposition in liquids. H_2_O_2_ was measured using the amplex ultra-red kit as described before [109] against a known standard of H_2_O_2_ and by reading the fluorescence at λ_ex_ 535 nm and λ_em_ 590 nm using a bandpass filter-based multimode reader (Tecan, Männedorf, Switzerland). This measurement and all plasma treatments were adjusted for water evaporating during plasma treatment using a fine-scale to predetermine the volume of double-distilled water added after each plasma treatment time to restore iso-osmotic conditions.

### 4.4. Metabolic Activity and Viability

Metabolic activity was analyzed by quantification and control-normalization of fluorescent resorufin 4 h after addition of resazurin (final concentration 100 µM; Alfa Aesar, Karlsruhe, Germany) at λ_ex_ = 535 nm and λ_em_ = 590 nm using a multimode reader (Tecan, Männedorf, Switzerland). For this assay, 1 × 10^4^ PDA cells were seeded one day before plasma treatment. As a control, the ROS scavenger n-acetylcysteine (NAC, final concentration: 2 mM; Sigma, Taufkirchen, Germany) was used. In a different setup, 7.5 × 10^4^ PDA cells were seeded in flat-bottom 24-well plates (NUNC, Roskilde, Denmark). One hour after plasma treatment, transwells were added to each well, and 7.5 × 10^4^ RAW cells were added into the transwell. Twenty-four hours later, the transwells were removed, and resazurin was added and analyzed as described above. For analysis of cell viability, 2 × 10^3^ PDA cells were added to each well of a 96-well plate, and propidium iodide (PI, final concentration 1 µg/mL; Merck, Darmstadt, Germany) was added. The cells were imaged (brightfield and PI) kinetically, and the number of viable cells against all cells was counted manually for each time point. 

### 4.5. Cell Migration and Quantitative Microscopy

The 2-well micro-insert (Ibidi, Gräfelfing, Germany) was added to the center of a well of 24-well plates. To each chamber, 1 × 10^4^ PDA cells were added and allowed to adhere overnight. Then, each side was plasma-treated, and the chamber was lifted to allow the analysis of cells migrating into the 400–500 µm-wide gap. Brightfield imaging was performed at 0 and 24 h using an inverted microscope at 200× magnification (Carl Zeiss, Jena, Germany). Cell migration within these 24 h was reconstructed using overlay images. The total growth area of untreated and plasma-treated PDA cells was analyzed using Harmony 4.9 imaging software (PerkinElmer, Hamburg, Germany). The cellular area was determined using digital phase-contrast imaging using an Operetta CLS high-content imaging device (PerkinElmer, Hamburg, Germany). This device and software were also used to determine mean speed during kinetic live-cell imaging of PDA cells, for which 2.5 × 10^3^ PDA cells were seeded, plasma-treated, and incubated for 1 h before the addition of 2.5 × 10^3^ RAW cells. For imaging of cluster formation induced by PDA-induced M2 macrophage polarization [99], 2 × 10^5^ PDA and RAW cells were seeded in wells of 6-well plates and imaged at 72 h using an Observer Z.1 (Carl Zeiss, Jena, Germany). Analysis of cluster numbers was performed using ImageJ software (Wayne Rusband, NIH; open source).

### 4.6. TUM-CAM Assay and Tumor Tissue Analysis

The in ovo TUM-CAM assay was performed as described before [87]. Briefly, the eggs were placed under a sterile workbench in a warming vessel (37 °C), carefully opened, and a silicon ring (diameter 0.5 cm) was placed on the CAM. Then, 2 × 10^6^ PDA6606 cells were suspended in Matrigel (Corning, Wiesbaden, Germany) and added into silicon rings embedded onto the CAM. Three days later, the tumors were plasma-treated manually for 60 s. 

### 4.7. In Ovo Tumor Tissue Analysis

Three days after plasma exposure, the eggs were put in egg-shaped, pre-warmed metal boxes and photographed using a 10× magnification objective of a stereo microscope to resolve vessel structures. For magnetic resonance imaging (MRI), eggs were cooled on ice for 5 min before data acquisition to inhibit chicken movement. The CAM was scanned in a high-field 7-Tesla MRI scanner for small animals (ClinScan, 7.0 T, 290 mT/m gradient strength, Bruker, Ettlingen, Germany). MRI scan analyses were performed in an egg coil filled with ice (Bruker, Ettlingen, Germany) using a T2-TSE (turbo-spin echo) sequence. For tumor size assessment, high-resolution T2-weighted images of the horizontal plane were used. Images were analyzed using MIPAV (medical imaging processing and visualization; National Institutes of Health, Bethesda, MD, USA). Afterward, tumors were separated from the CAM with surgical scissors and weighted. Some tumors were subsequently cryopreserved and sectioned using a cryotome (ThermoFisher, Dreieich, Germany). The 6 µm-thick sections were fixed in paraformaldehyde (4%) and permeabilized with 0.1% Triton X-100 (ThermoFisher, Dreieich, Germany). Apoptotic cells were visualized using a TdT-mediated dUTP-biotin nick end labeling (TUNEL) kit (R&D Systems, Wiesbaden, Germany) according to the manufacturer’s instructions. Nuclei were counterstained using 4′6-diamidino-2-phenylindole (DAPI, final concentration 1 µM; Sigma, Taufkirchen, Germany). Fluorescence microscopy was performed using a BZ-9000 device (Keyence, Osaka, Japan) with an exposure time of 10 ms and with the BZ-II-Analyzer 4.6.2.2. Software (Keyence, Osaka, Japan).

### 4.8. Flow Cytometry

For multicolor flow cytometry of PDA-RAW co-cultures at 24 and 96 h post-plasma treatment, supernatants were harvested, centrifuged, and these supernatants were collected and stored at −20 °C for cytokine and chemokine analysis. The adherent cells were added to the previously detached pellets using accutase (BioLegend, Amsterdam, The Netherlands). The cells were washed with phosphate-buffered saline (PBS) and incubated with fluorescently labeled monoclonal antibodies targeting CD11b, CD115, CD206, and Ly6c (all BioLegend, Amsterdam, The Netherlands). Subsequently, cells were washed and acquired using a CytoFLEX S device (Beckman-Coulter, Krefeld, Germany). Analysis was performed using Kaluza 2.1 software (Beckman-Coulter, Krefeld, Germany). Multiplex chemokine and cytokine analyses were performed using LEGENDplex (BioLegend, Amsterdam, Netherlands), as described before [110]. Briefly, the supernatants were incubated with antibody-coated capture beads that, together with an amplification system, showed a concentration-dependent intensity when analyzing the beads by flow cytometry and employing 5-log functions in dedicated software 8 (Vigenetech, Carlisle, MA, USA) for calculating absolute target concentrations.

### 4.9. ELISA

To quantify vascular endothelial growth factor (VEGF), a commercially available ELISA kit was utilized according to the manufacturer’s instructions (BioLegend, Amsterdam, The Netherlands). Absorption was measured on an M200 multimode plate reader (Tecan, Männedorf, Switzerland) against a known standard.

### 4.10. Statistical Analyses

Statistical analysis was performed using prism 9.1 (GraphPad Software, San Diego, CA, USA). Data are representative or mean of three experiments, if not indicated otherwise. The statistical comparison between several groups was determined with a one-way analysis of variance (ANOVA). If all groups were tested against a single control group, Dunnett’s post-hoc test was performed. To compare the means of two groups, a two-sided Student’s *t*-test was performed. A *p*-value smaller than 0.05 was considered statistically significant (*), with ** and *** denoting *p* < 0.01 and *p* < 0.001, respectively. Arrows in chemokine and cytokine analysis indicate significant trends for increase or decrease as analyzed using one-way ANOVA.

## 5. Conclusions

Gas plasma treatment targeting murine pancreatic cancer cells modulated their secretion products and recognition, as well as inflammatory conditioning by murine macrophages that did not follow the classical M1/M2 pattern. These results suggest that plasma-induced cell death is accompanied by a modulation of the immuno-controlled tumor microenvironment, which might play a role in the future plasma treatment of cancer in medical settings. 

## Data Availability

The data presented in this study are available on request from the corresponding author.
